# Photocatalytic valorization of lignin: radical-mediated scission of recalcitrant bonds to aromatics

**DOI:** 10.1039/d5ra04043d

**Published:** 2025-07-02

**Authors:** Fang-Fang Tan

**Affiliations:** a School of Chemistry and Materials, Weinan Normal University 714099 Weinan Shaanxi China tanfangfang2021@outlook.com

## Abstract

Lignin, a three-dimensional aromatic polymer formed by the cross-linking of arylpropanol units *via* C–C/C–O bonds, faces the critical scientific challenge of achieving selective cleavage of these bonds to enable its valorization and the directional synthesis of renewable aromatic compounds. Photocatalysis offers a novel strategy for targeted bond dissociation through precise regulation of electron transfer pathways and radical generation modes. This review systematically elucidates the mechanisms underlying photocatalytic C–C and C–O bond cleavage in lignin systems. For C–C bond cleavage, three primary pathways include: generation of oxygen-centered radicals *via* ligand-to-metal charge transfer (LMCT) or proton-coupled electron transfer (PCET) processes, inducing β–C–C bond cleavage; generation of carbon-centered radicals *via* hydrogen atom transfer (HAT) or single-electron transfer (SET) processes, followed by C–C bond cleavage *via* oxygen participation. For C–O bond cleavage, the main pathways are: a stepwise oxidation-reduction mechanism driven by HAT or SET; generation of carbon-centered radicals *via* HAT or SET, inducing β–C–O bond cleavage; or activation of lignin models or auxiliary reagents *via* SET to form reactive radicals inducing C–O bond cleavage. These pathways universally rely on photocatalytically generated radicals (*e.g.*, oxygen/carbon-centered radicals), which redistribute electrons and significantly weaken β–C–C and β–C–O bonds. Based on these insights, we propose feasible strategies for efficient native lignin depolymerization through catalyst electronic structure optimization and reaction microenvironment modulation, providing a theoretical framework for the photoelectrocatalytic valorization of lignin resources.

## Abstract 2

This review summarizes visible-light-driven radical cleavage of lignin C–C/C–O bonds for efficient valorization under mild conditions.

## Graphical abstract

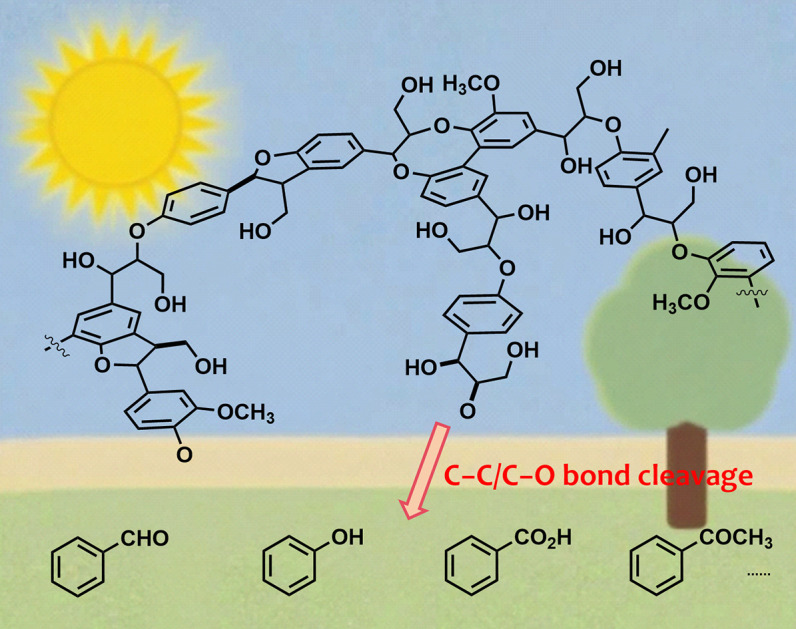

## 1. Introduction

**Fig. 1:**
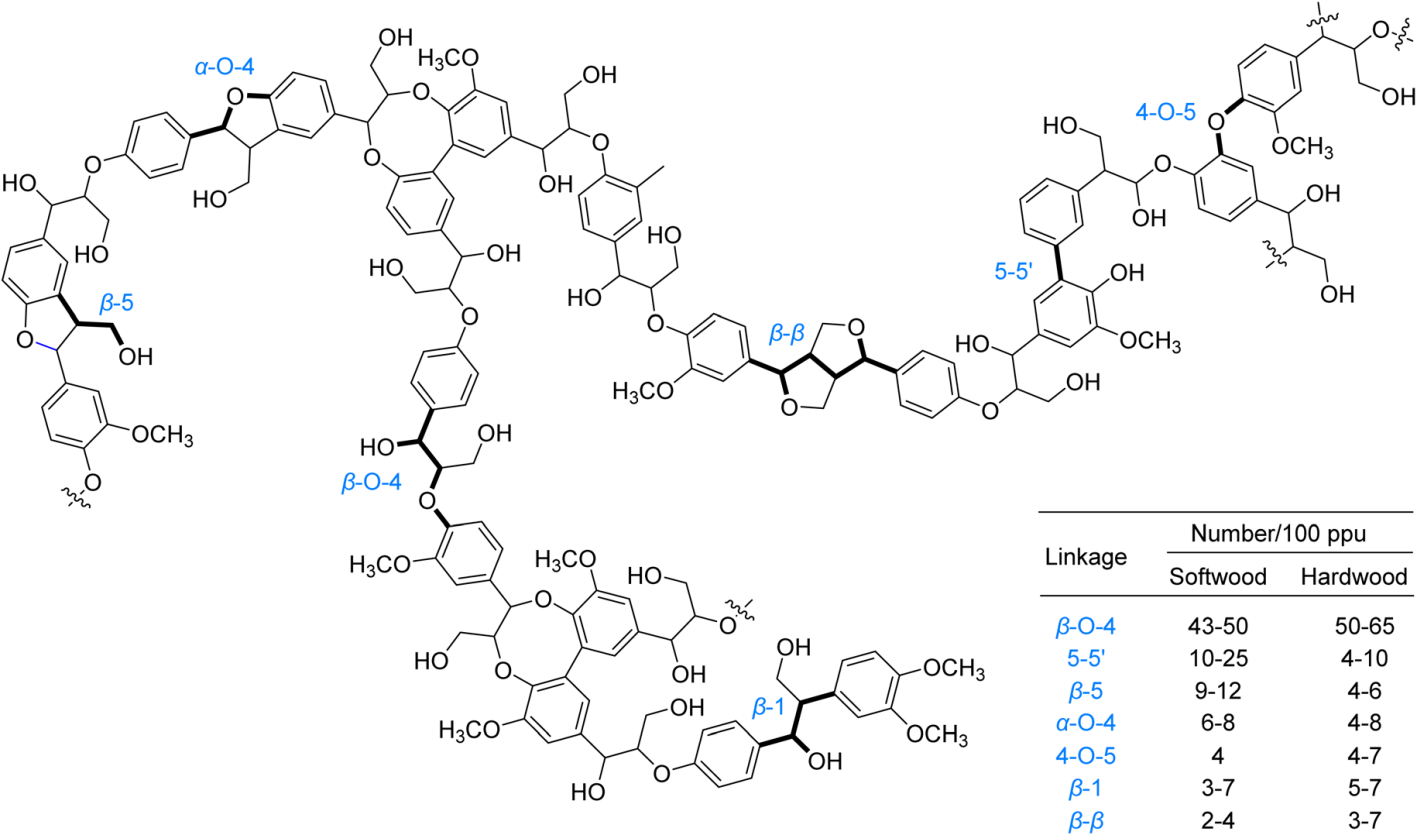
Lignin structural fragments and distribution of linkage types.

Lignin, the second most abundant biopolymer on Earth, constitutes approximately 30% of lignocellulosic biomass by weight and serves as the primary natural source of aromatic compounds.^1–8^ Its three-dimensional network structure arises from the cross-linking of phenylpropane units through various inter-unit linkages, such as β–O–4, 4–O–5, 5–5′, and β-β, among which the β–O–4 bond predominates with an abundance of 43–65% (Fig. 1).^2,4,7^ This complex macromolecular structure provides structural rigidity to plant cell walls and confers significant biological recalcitrance. However, the recalcitrance of these inter-unit linkages, particularly the high bond dissociation energies of C–C and C–O bonds, presents a major challenge for efficient valorization.^1–8^ Conventional thermocatalytic depolymerization strategies typically require harsh conditions, including high temperatures and pressures, which often lead to uncontrolled bond cleavage, undesired repolymerization and dearylation side reactions, and unsatisfactory yields of valuable aromatic compounds.^9–18^ Currently, over 95% of industrial lignin is combusted for low-grade energy recovery, revealing a stark gap between its intrinsic potential as a renewable carbon feedstock and its persistently suboptimal valorization efficiency within sustainable chemical syntheses.^1,3,5–7^

**Fig. 2:**
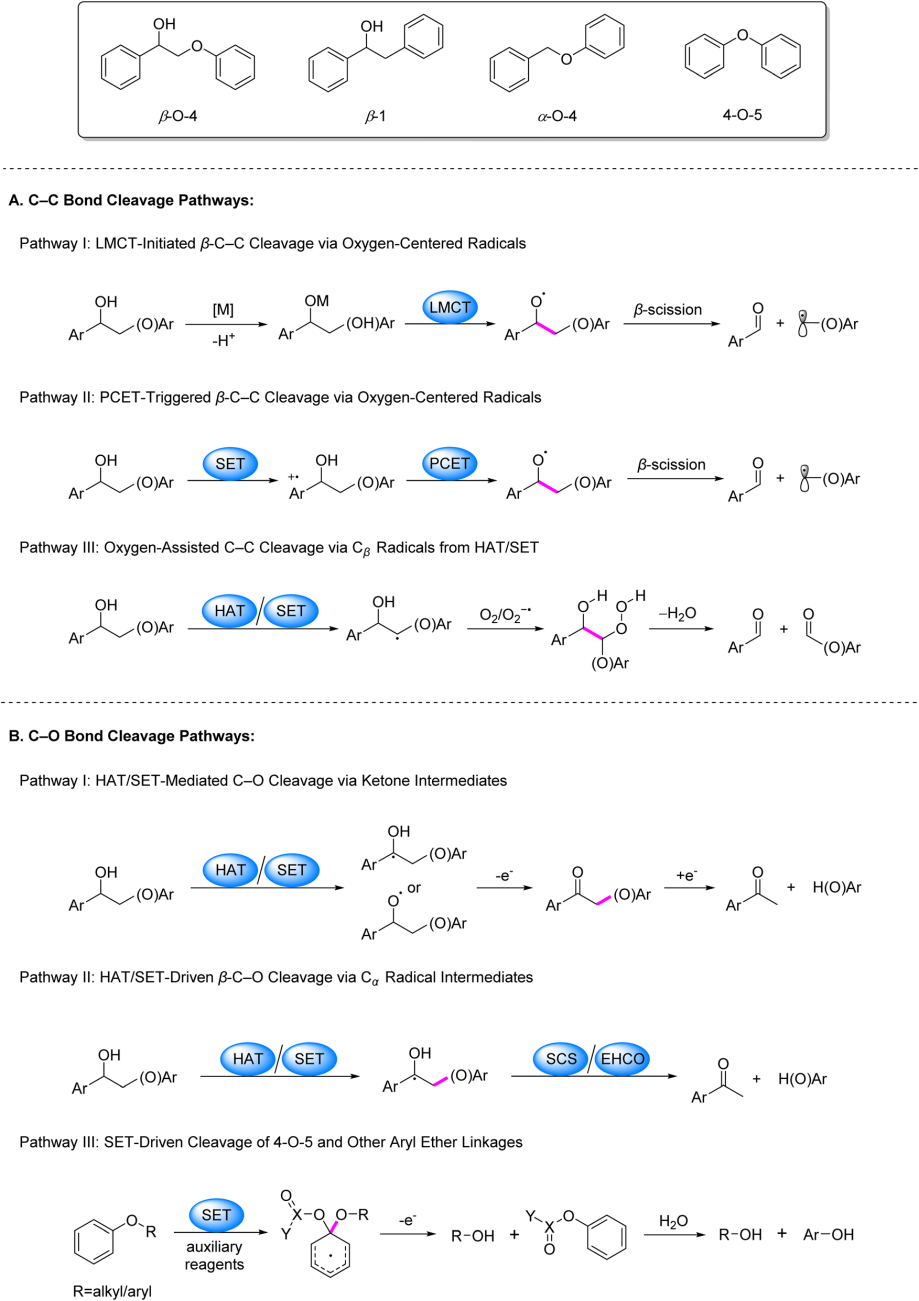
Dominant pathways of C–C and C–O bond cleavage in lignin under photocatalytic conditions.

Photocatalysis has emerged as a powerful strategy for biomass valorization under mild conditions, leveraging the generation of carbon- or oxygen-centered radicals to attenuate bond energies of β–C–C/C–O linkages and drive selective bond cleavage.^3,4,19^ Recent advancements in this field predominantly focus on lignin model systems, particularly β–O–4, β-1, α–O–4 and 4–O–5 linkages, encompassing both C–C and C–O bond activation. The prevailing pathways for C–C bond activation are categorized as follows (Fig. 2): (1) coordination of alcoholic hydroxyl groups in model compounds with metal ions facilitates LMCT under irradiation, generating oxygen-centered radicals that reduce β–C–C bond energy and induce cleavage (Fig. 2A, Pathway I); (2) single-electron oxidation of model molecules by photocatalysts produces aryl cation radicals, which undergo intramolecular PCET in the presence of base to form oxygen-centered radicals, thereby weakening β–C–C bonds (Fig. 2A, Pathway II); (3) formation of C_β_ radicals *via* SET or HAT processes, followed by their reaction with O_2_/O_2_^−^˙ to generate peroxide intermediates that undergo homolytic C–C bond scission (Fig. 2A, Pathway III).

Regarding C–O bond activation, primary mechanisms include: (1) the generation of C_α_ or oxygen-centered radicals *via* SET or HAT, followed by oxidation to ketone intermediates and subsequent reductive cleavage (Fig. 2B, Pathway I); (2) cleavage of the β–C–O bond triggered either by a spin center shift (SCS) process following the generation of a C_α_ radical *via* HAT, or by electron–hole coupling (EHCO) on heterogeneous systerms (Fig. 2B, Pathway II); for 4–O–5 and α–O–4 models, (3) SET between substrates/auxiliary reagents and photocatalysts to generate dearomatized radicals that promote C–O bond cleavage (Fig. 2B, Pathway III).

The elucidation of these diverse radical-mediated pathways, each capable of selectively weakening critical C–C and C–O bonds, represents significant progress in understanding photocatalytic lignin depolymerization. Building upon this foundation, this review critically examines recent progress in photocatalytic lignin valorization. We further propose that rational catalyst design, focusing on electronic structure optimization and reaction microenvironment modulation, represents a feasible strategy towards achieving efficient and selective depolymerization of native lignin under mild conditions. Ultimately, this work aims to consolidate the mechanistic framework and emerging strategies to bridge the gap between lignin's immense potential as a renewable aromatic resource and its practical, sustainable utilization.

## 2. Photocatalytic C–C bond cleavage in lignin: three pathways and mechanisms

### 2.1 LMCT-initiated β–C–C cleavage *via* oxygen-centered radicals

**Fig. 3:**
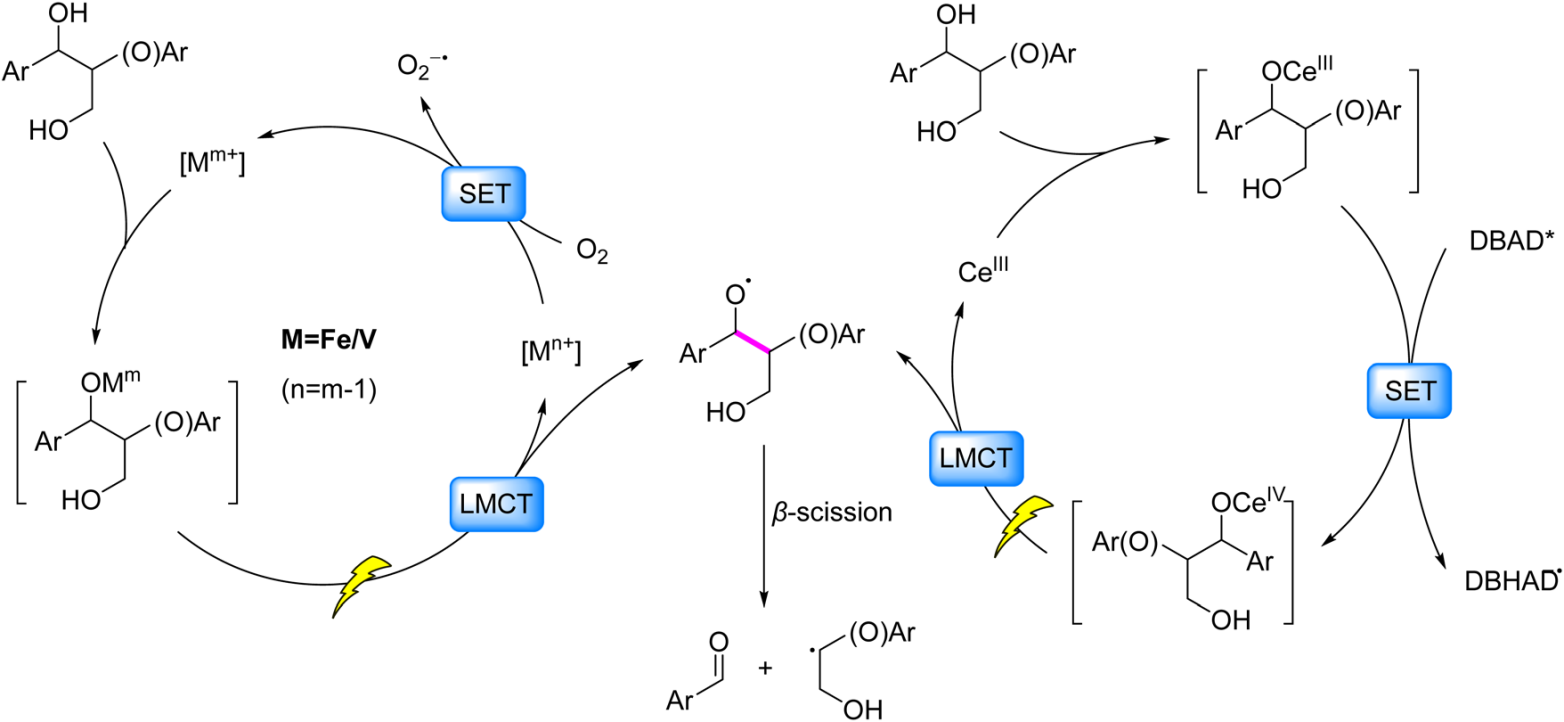
LMCT-mediated formation of oxygen-centered radicals inducing β–C–C scission.

The LMCT mechanism involves electron transfer from ligand-based orbitals to vacant metal-centered orbitals under light irradiation, enabling the formation of oxygen-centered radicals.^20^ This process is particularly effective in coordination complexes featuring electrophilic high-valent metal centers (*e.g.*, Fe(iii), V(v), Ce(iv)), where the low energy barrier facilitates electron transfer from electron-rich ligands (*e.g.*, halides, carboxylates) to empty metal d-orbitals.^20^ σ- or σ/π-donor ligands further enhance LMCT transitions at reduced energy thresholds, providing a strategic pathway for selective C–C bond activation in lignin. This mechanistic foundation has driven extensive exploration of LMCT-based strategies for C–C bond cleavage in β–O–4 model compounds during lignin valorization. A generalized pathway involves coordination of lignin-derived alcohols to transition metals (*e.g.*, V(v), Fe(iii)), followed by light-induced LMCT to generate alkoxy radicals that significantly reduce the BDE of β–C–C bonds, ultimately leading to their selective cleavage (Fig. 3). However, the process typically requires oxidizing agents (*e.g.*, O_2_) to regenerate the metals to their original oxidation states, which introduces challenges in energy efficiency and reaction sustainability.

The historical progression of this field originates in the seminal work by Aliwi *et al.* (1975),^21^ who first documented homolytic cleavage of V(v)–OMe bonds under 365 nm irradiation, generating methoxy radicals through a V(v)-centered LMCT pathway. Gazi *et al.* (2015)^22^ advanced this approach using a hydrazoneamide-ligated vanadium complex under visible light, achieving C_α_–C_β_ bond cleavage in β–O–4 model compounds to afford aryl aldehydes and formates, albeit with limited efficiency (<40% combined yield). Subsequent ligand engineering (*e.g.*, fluoro/nitro functionalization) by the same group^23^ in 2017 markedly improved yields to 72% for benzaldehyde and 35% for aryl formates, underscoring the critical role of ligand design in catalytic performance. Liu *et al.* (2019)^24^ further demonstrated commercial VO(i-Pr)_3_-catalyzed β–O–4 cleavage under O_2_ atmosphere, producing diverse products (aryl aldehydes, phenols, carboxylic acids) with 1.39% monomer yield from native birch lignin. Zhang *et al.* (2021)^25^ expanded the scope of LMCT-driven depolymerization *via* an Fe(iii)/Ce(iii) bimetallic system under aerobic conditions, attaining 84% benzoic acid yield from β-O-4 models while phenyl formate remained a minor byproduct (22%), highlighting both the versatility of Fe-based LMCT catalysis and persistent selectivity challenges. Most recently, Liu *et al.* (2024)^26^ reported FeCl_3_/NaCl or FeCl_3_/nBu_4_Cl-catalyzed LMCT activation at 390 nm, cleaving β-O-4 and β-1 model C–C bonds to deliver benzaldehyde (∼90%) and N-heterocycles (98%), with this protocol converting solvent-fractionated lignin into two primary aryl aldehydes at 8.7% combined yield.

The pursuit of cost-effective catalysts has significantly broadened the application scope of LMCT in lignin depolymerization. Cerium(iii) chloride (CeCl_3_), a commercially accessible Lewis acid, has emerged as a pivotal catalyst in LMCT-driven systems. Guo *et al.* (2016)^27^ pioneered a Ce(iii)-alcohol coordination complex that undergoes photoinduced LMCT, generating alkoxy radicals to cleave β-C–C bonds with di-*tert*-butyl azodicarboxylate (DBAD) as an electron acceptor. This foundational work established the mechanistic framework for subsequent refinements by Wang *et al.* (2020)^28^ (Fig. 3). Their advanced strategy achieved 97% aryl aldehyde yield from β-O-4 and β-1 model compounds, alongside an 11.94% monomer yield from native pine lignin.

Several persistent challenges remain to be addressed in current LMCT-based systems. First, the requirement for stoichiometric oxidants or electron acceptors to regenerate the metal catalysts to their initial oxidation states often triggers overoxidation of products, consequently compromising selectivity. Furthermore, the reliance on specific electron acceptors inherently restricts the diversity of applicable reactions. Second, the abundance of hydroxyl groups within native lignin architectures may introduce competing side reactions that diminish selectivity during practical degradation processes. Most critically, achieving efficient depolymerization of native lignin remains a formidable challenge for existing LMCT systems, primarily due to the inherent difficulty in selectively activating the recalcitrant cross-linked bonds within its three-dimensional polymeric network.

### 2.2 PCET-triggered β-C–C cleavage *via* oxygen-centered radicals

**Fig. 4:**
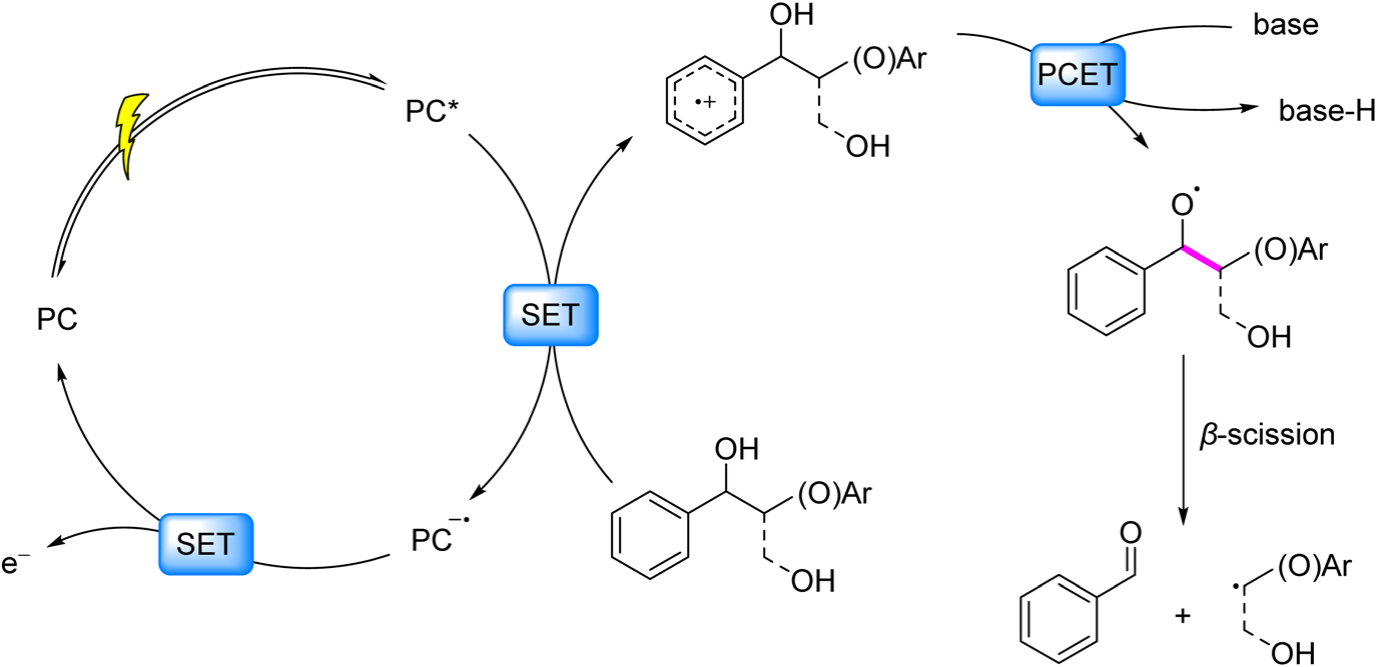
Photoinduced generation of oxygen-centered radicals *via* PCET process.

The PCET mechanism provides thermodynamic advantages by mitigating charge accumulation at individual sites while kinetically bypassing high-energy intermediates.^29^ In this concerted process, an oxidant and a Brønsted base synergistically remove one electron and one proton, respectively, from substrates. The independent tunability of oxidant/base pairs enables precise modulation of PCET energetics, facilitating efficient cleavage of high-bond-dissociation-energy linkages. This framework has been rigorously validated through combined spectroscopic and mechanistic studies. For aryl alcohols, time-resolved spectroscopy by Bacchioici, Bietti, and Steenken^30–32^ revealed diffusion-controlled deprotonation of benzylic C–OH groups adjacent to aryl radical cations, generating alkoxy radicals. Knowles *et al.*^33^ established the concerted nature of this PCET *via* kinetic isotope effects, establishing a cornerstone mechanism for C_α_–C_β_ cleavage in lignin β-O-4 and β-1 linkages. The catalytic cycle comprises three steps: photooxidation of arenes to radical cations, base-assisted benzylic deprotonation forming alkoxy radicals, and β-scission driven by adjacent bond weakening (Fig. 4).

Translating this mechanistic framework into synthetic applications, Ota *et al.* (2019)^34^ implemented PCET-driven C_α_–C_β_ cleavage using an [Ir] photocatalyst paired with tetrabutylphosphonium dimethoxyphosphate (PBu_4_OP(O)(MeO)_2_) as a Brønsted base under visible light. This system achieved 89% aryl aldehyde and 65% aryl ether yields from β-O-4 lignin models. Parallel efforts by Wang *et al.*^35^ employed an identical [Ir] photocatalyst with 2,4,6-trimethylpyridine (collidine), extending substrate scope to β-O-4 and β-1 linkages with 94% aldehyde and 93% ether yields. A critical limitation inherent to native lignin systems is the competitive participation of ubiquitous phenolic hydroxyl groups in PCET processes, which suppresses the activation of aliphatic C_α_–OH motifs. To overcome this selectivity barrier, Wang *et al.* developed a pretreatment protocol utilizing CH_3_I/K_2_CO_3_ to protect phenolic OH groups. This approach yielded 4.99% monomeric aromatics from birch lignin. Nguyen *et al.*^36^ leveraged the BDE differential between phenolic and aliphatic O–H bonds. Their advanced catalytic system integrated an [Ir] photocatalyst, the proton acceptor PBu_4_OP(O)(OtBu)_2_, and 2,4,6-triisopropylthiophenol (TRIP-SH) as a HAT mediator. This system enabled the direct depolymerization of organosolv lignin, achieving a monomer yield of 5.1% from pine lignin.

Zhou *et al.* (2018)^37^ developed a visible-light-driven photocatalytic system utilizing an acridinium salt as the photocatalyst and NaOAc as a base. This system enabled selective C_α_–C_β_ bond cleavage in β-O-4 and β-1 lignin model compounds, producing aryl aldehydes (∼71%), aryl formates (∼58%), and HCHO. Crucially, molecular oxygen served as an electron acceptor to regenerate the ground state of the photocatalyst. The *in situ* generation of peroxides or O_2_-mediated oxidation not only closed the catalytic cycle but also led to oxygenated byproducts, underscoring the versatility of oxidant-coupled strategies. Further advancing radical utilization, Patehebieke *et al.* (2023)^38^ reported utilizing an acridinium photocatalyst with collidine as the base to cleave the C–C bond in β-O-4 lignin models *via* a PCET process. The resulting carbon-centered radicals underwent efficient addition to olefins, affording radical adducts in ∼64% yield. This strategy was extended by Li *et al.*,^39^ demonstrating radical trapping by azo compounds to access nitrogen-containing derivatives. Collectively, these studies highlight the potential of engineered radical intermediates to diversify product profiles while maintaining catalytic efficiency.

Recent innovations continue to refine PCET-based cleavage. Rao *et al.* (2024)^40^ developed an efficient pyridinium-based photocatalytic system for the selective cleavage of the C_α_–C_β_ bond in β-O-4 and β-1 lignin model compounds. The strategy leverages the strong electron-accepting capability of the pyridinium core, synergistically coupled with the neutral nitrogen atom within a pyridazine substituent, to establish a PCET pathway. This mechanism facilitates hydrogen abstraction from the substrate's hydroxyl group, generating radical intermediates that directly induce β-C–C bond scission, predominantly yielding benzaldehyde and phenyl formate as the primary products. Concurrently, Xu *et al.*^41^ reported efficient photocatalytic cleavage of C_α_–C_β_ bonds in lignin β-1 models using a cyanide/potassium-modified graphitic carbon nitride photocatalyst (6MCN) in aqueous solvent, achieving yields of 46.3% for benzaldehyde and 38.4% for benzoic acid. Most recently, Zheng *et al.* (2024)^42^ introduced a bifunctional 2-bromoanthraquinone (2-Br-AQN) photocatalyst. Under 405 nm light, it cleaves β-O-4 models *via* PCET, delivering high yields of aryl aldehydes (∼92%) and phenols (∼87%). Critically, mechanistic studies confirmed excited-state proton/electron abstraction forms an oxygen-centered radical triggering β-scission. Demonstrating practical relevance, this method achieved 2.35–5.37% aryl monomer yields from native lignins (pine, fir, poplar).

### 2.3 Oxygen-assisted C–C cleavage *via* C_β_ radicals from HAT/SET

**Fig. 5:**
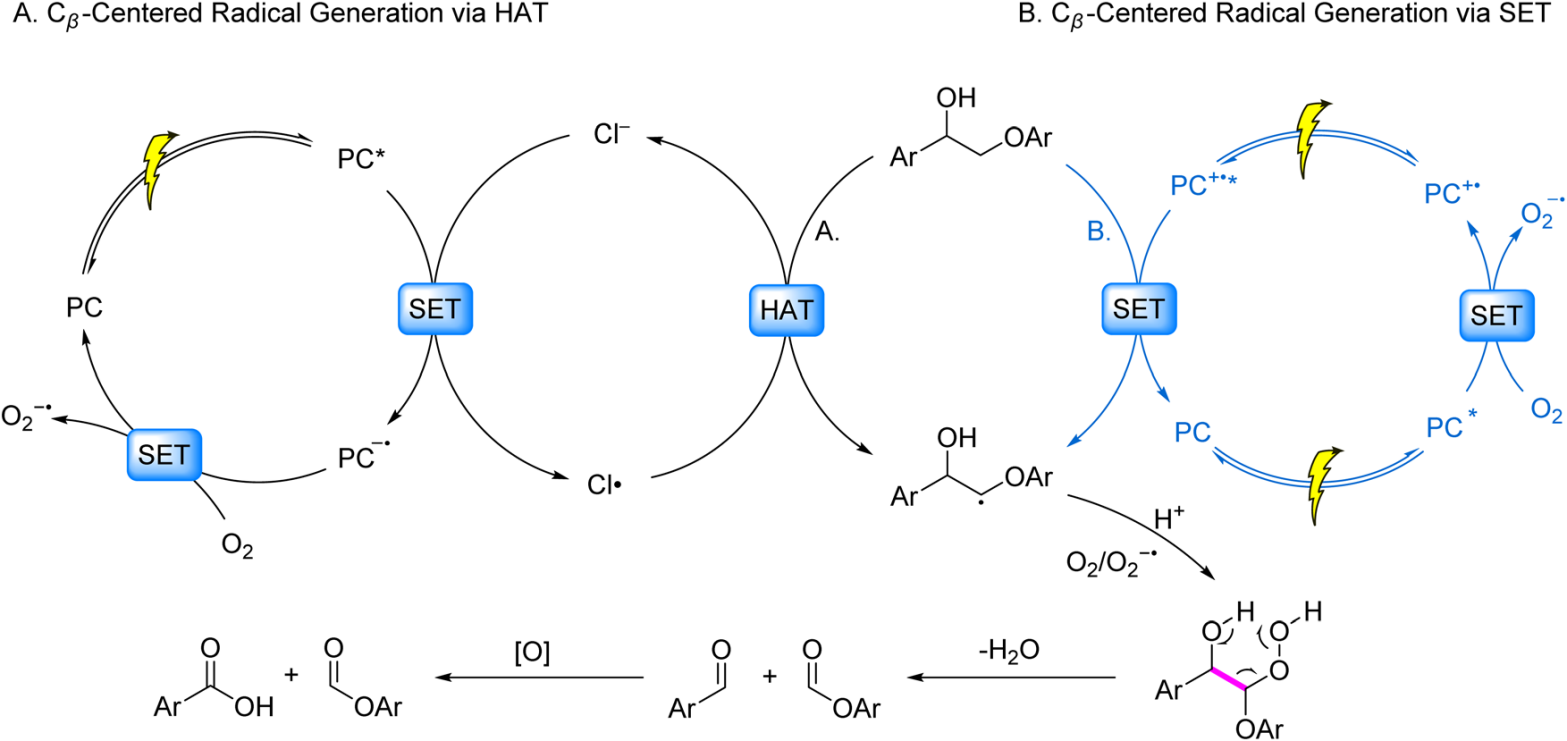
Mechanisms of β-C–C bond cleavage induced by C_β_-centered radicals generated *via* HAT/SET.

The oxidative cleavage of lignin C–C bonds through oxygen-mediated pathways can be mechanistically rationalized by two complementary strategies involving HAT and SET processes (Fig. 5). These approaches synergistically exploit reactive oxygen species (ROS) to achieve β-bond destabilization and subsequent bond scission. The HAT-driven mechanism^43^ initiates with the abstraction of a hydrogen atom from the C_β_–H bond by a chlorine radical (Cl˙), a prototypical HAT reagent. The Cl˙ species is generated *in situ via* SET between chloride ions (Cl^−^) and a photocatalyst under light irradiation. This hydrogen abstraction directly yields a C_β_ radical, which subsequently participates in oxygen–mediated bond cleavage (Fig. 5A). Alternatively, the SET-dominated route involves direct interaction between the photocatalyst and the lignin substrate. Single-electron oxidation of the C_β_–H bond produces a cationic radical intermediate. Rapid deprotonation of the activated acidic proton then liberates the C_β_ radical, priming it for oxidative transformation (Fig. 5B). Both pathways converge at the critical C_β_ radical intermediate, which reacts with O_2_/O_2_^−^˙ to form a six-membered cyclic peroxide transition state. The metastable peroxide undergoes spontaneous dehydration, ultimately triggering C–C bond cleavage and generating two carbonyl-terminated fragments.

The HAT process has been effectively integrated into photocatalytic systems for selective C–C bond cleavage in lignin, demonstrating remarkable versatility and efficiency. Li *et al.* (2023)^44^ developed a synergistic photocatalytic system combining perylene diimide (PDI) as an organic photosensitizer with tetrabutylammonium chloride (*n*Bu_4_NCl) as a HAT catalyst under visible light irradiation and aerobic conditions. This system achieved selective cleavage of C_α_–C_β_ bonds in both β-O-4 and β-1 lignin model compounds, producing aryl aldehydes (21% yield), aryl carboxylic acids (70% yield), and aryl formates (74% yield) (Fig. 5A). Building on this work, Xu *et al.* (2024)^45^ employed commercially available 2,4,6-triphenylpyrylium tetrafluoroborate (TPT) as a photocatalyst with calcium chloride (CaCl_2_) as a HAT catalyst under oxygen atmosphere. Their system demonstrated efficient C_α_–C_β_ bond cleavage in β-O-4 model compounds, yielding aryl carboxylic acids (77%) and aryl formates (76%) (Fig. 5A). Notably, this methodology was successfully applied to solvent-fractionated lignins derived from birch and pine, achieving aromatic monomer yields of 0.69 wt% and 0.42 wt%, respectively.

Complementary to HAT-dominant systems, SET mechanisms have been advanced through innovative dual-photon excitation strategies. In a seminal study, Li *et al.*^46^ employed phenothiazine (PTH) as a dual-photon photocatalyst under 390 nm irradiation and aerobic conditions, achieving selective cleavage of β-O-4 and β-1 model compounds to afford aryl aldehydes and carboxylic acids. Transient absorption spectroscopy revealed a stepwise excitation mechanism: initial photoexcitation generates the excited state (PTH*), which undergoes oxidative quenching by O_2_ to yield PTH^+^˙ and O_2_^−^˙. The singly oxidized species PTH^+^˙ (*E*_ox_ = +1.45 V *vs.* NHE) exhibits insufficient potential for direct C_β_–H bond activation. Subsequent absorption of a second photon generates the doubly excited state PTH^+^˙* (*E*_ox_ = +2.31 V *vs.* NHE), enabling efficient SET-mediated C_β_–H oxidation. The resultant C_β_-centered radical undergoes trapping by O_2_^−^˙, forming a transient cyclic peroxide intermediate that facilitates β-scission to release benzaldehyde derivatives (Fig. 5B). Notably, competitive C_α_–H overoxidation pathways were identified, generating ketonic byproducts *via* secondary SET processes that form C_α_ radicals. This mechanistic dichotomy highlights the critical balance between selective C_β_ activation and undesired side reactions in photocatalytic transformations. In parallel, Wang^47^ and Li *et al.*^48^ have independently reported the photoinduced cleavage of C_α_–C_β_ bonds in lignin β-O-4 and β-1 models using Mes-Acr^+^–BF_4_^−^ and TPT as photocatalysts, respectively. This transformation proceeds *via* a SET-mediated mechanism with oxygen assistance, yielding aryl aldehydes, ketones, and other aryl derivatives. Subsequent oxidation of the aryl aldehydes under these strongly oxidative conditions typically generates aryl carboxylic acids as the predominant products.

**Fig. 6:**
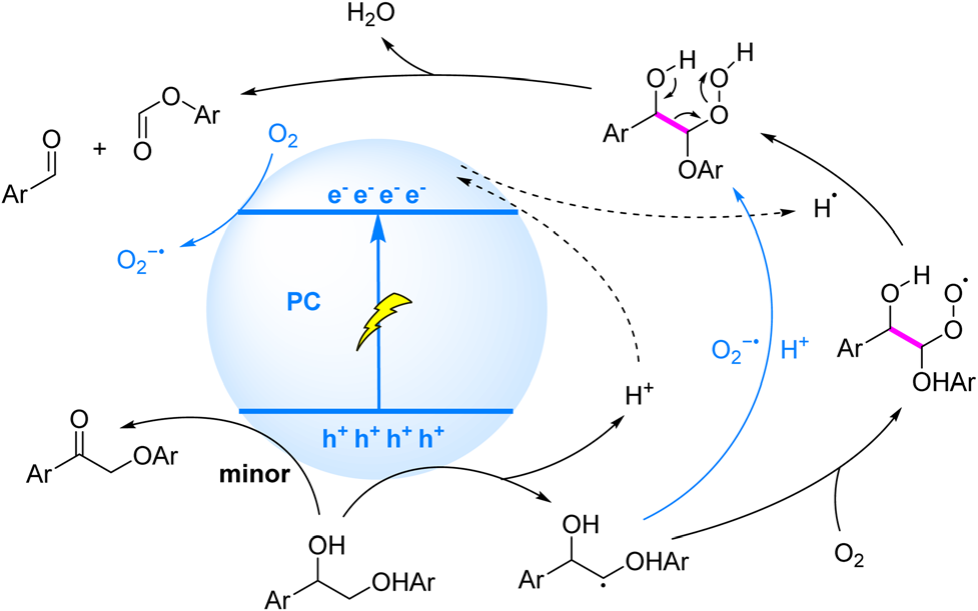
Mechanism of β-C–C bond cleavage *via* generation of C_β_-centered radicals in heterogeneous systems.

Furthermore, select semiconductor materials leverage their engineered defect structures to generate highly oxidizing holes (h^+^) through efficient charge separation under visible-light irradiation. These holes drive single-electron oxidation of β-O-4 and β-1 lignin model compounds, forming C_β_-centered radicals that facilitate C–C bond cleavage *via* a homogeneous-like mechanism (Fig. 6). Representative studies have progressively advanced the photocatalytic cleavage of lignin model compounds through tailored material design. Liu *et al.* (2018)^49^ demonstrated mesoporous graphitic carbon nitride (mpg-C_3_N_4_) efficiently cleaves C–C bonds in β-O-4 and β-1 models under 455 nm LED irradiation, yielding aryl aldehydes (51%), aryl carboxylic acids (21%), and formate phenolic esters (30%), with ketones (7%) identified as hydroxyl oxidation byproducts. The material's extended π-conjugation facilitates strong π–π stacking interactions with lignin substrates, enabling precise directional activation of C_β_–H bonds by photogenerated holes. Wu *et al.* (2021)^50^ developed a Z-scheme Ag_3_PO_4_/polymeric carbon nitride (PCN) nanocomposite that achieves efficient lignin C–C bond cleavage under simulated sunlight at ambient temperature, converting diverse model compounds to aromatic aldehydes in 66–95% yields. The Z-scheme configuration preserves the strong redox capabilities of both components, enabling synergistic electron–hole participation in C_α_–C_β_ bond scission. Wang *et al.* (2022)^51^ reported a polyimide-based photocatalyst (PI-BT) that achieves near-quantitative cleavage (>99% conversion, >99% selectivity) of the C_α_–C_β_ bond in β-O-4 models under visible light in air. This performance stems from PI-BT's optimized electronic structure, which enhances hole extraction efficiency and radical stability while suppressing over-oxidation side reactions common in homogeneous systems. Chen *et al.* (2022)^52^ synthesized sea-urchin-like Nb_2_O_5_ hollow microspheres (U–Nb_2_O_5_ HM) *via* a one-pot hydrothermal method, achieving highly selective C_α_–C_β_ bond cleavage (94% conversion, 96% selectivity) in β-O-4 models under mild conditions. Combined experimental and DFT studies revealed that exposed active sites, efficient carrier separation, and reactive (001) facets collectively promote C_β_–H activation, where photogenerated holes initiate critical C_β_ radical formation to drive bond scission.

Building upon foundational systems, recent material innovations leverage strategic modifications to overcome persistent limitations in photocatalytic efficiency and selectivity. Xu *et al.* (2023)^53^ engineered nitrogen-doped graphitic carbon nitride (15DCN) *via* DMF-mediated synthesis, achieving 100% C–C bond cleavage efficiency through enhanced light harvesting and carrier separation, with benzaldehyde/benzoic acid as dominant products. Complementing doping strategies, Zhang *et al.* (2023)^54^ developed triazine-heptazine carbon nitride heterojunctions whose superior C–C cleavage activity over single-component systems arises from interfacial built-in electric fields accelerating charge separation and C_β_ radical generation. Notably expanding the materials toolkit, Lee *et al.* (2023)^55^ demonstrated that polyoxometalate Mo132 enables visible-light-driven aqueous-phase cleavage of β-O-4 C_α_–C_β_ bonds. The addition of K_2_S_2_O_8_ dramatically increased benzoate ester yield from 8.8% to 55.9% (95.7% selectivity) *via* a dual-path mechanism: holes oxidize C_α_–OH through LMCT to form carbon radicals, while electrons reduce O_2_ to O_2_^−^˙ for synergistic C_β_–H activation, with *in situ* generated H_2_O_2_ triggering Baeyer–Villiger-like homolytic cleavage.

The year 2024 witnessed paradigm-shifting breakthroughs in photocatalytic C–C cleavage, marked by sophisticated structural innovations that transcend conventional modification approaches. Cao *et al.*^56^ engineered multi-defect ultrathin g-C_3_N_4_ to optimize electronic structure and carrier dynamics, significantly boosting activity/selectivity. Advancing doping strategies, Duan *et al.*^57^ developed S and Cl co-doped triazine/heptazine carbon nitride (tri/hep-CN) enabling efficient β-1 cleavage in air, producing benzaldehyde/benzyl alcohol through enhanced carrier separation and elevated valence band potential. In the domain of heterojunction design, Jiang *et al.*^58^ constructed an S-scheme Cs_3_Bi_2_Br_9_/Bi_2_WO_6_ system where interfacial electric fields amplify light harvesting and charge separation, achieving 76–88% C_α_–C_β_ cleavage yields. Progressing beyond binary systems, Jiang *et al.*^59^ implemented a ternary Z-scheme g-C_3_N_4_/CQDs/WO_3_ heterojunction attaining 99% conversion/selectivity for β-O-4 cleavage (83.92% combined aromatic yield), with carbon quantum dots extending photoresponse and facilitating interfacial charge transfer. Simultaneously exploring hybrid architectures, Shi *et al.*^60^ embedded Cs_3_Bi_2_Br_9_ quantum dots in covalent triazine frameworks, achieving complete β-O-4 conversion through electronic structure-mediated radical control. Converging material and process innovation, Xu *et al.*^61^ synergized cyano-modified carbon nitride (KLCN) with persulfate oxidants for effective scission in β-1 models and diols, leveraging anisotropic morphology for optimized carrier transport.

## 3. Photocatalytic C–O bond cleavage in lignin: three pathways and mechanisms

### 3.1 HAT/SET-mediated C–O cleavage *via* ketone intermediates

Photocatalytic C–O bond cleavage *via* HAT/SET mechanisms fundamentally relies on carbon-centered radical generation and manipulation to drive thermodynamically favored scission. In β-O-4 model systems, this process initiates through C_α_–H bond activation forming a C_α_-centered radical, where subsequent oxidation generates a ketonic intermediate that reduces the C_β_–O BDE from 55 kcal mol^−1^ to 47 kcal mol^−1^.^62,63^ Crucially, stabilization of the C_α_ radical prior to oxidation further lowers the C_β_–O BDE to 7.8 kcal mol^−1^*via* conjugated electronic effects, establishing an intrinsic driving force for spontaneous bond cleavage. Early homogeneous catalytic systems, however, faced critical limitations in directly exploiting this low-BDE pathway due to poor selectivity activation sites coupled with rapid radical quenching by solvents or molecular oxygen. To circumvent these constraints, sequential oxidation-reduction strategies emerged: initial oxidative formation of ketonic intermediates under mild conditions precedes reductive cleavage of the electronically activated C_β_–O bond.^12,64–71^ Recent advances now enable synergistic integration of both steps within unified photocatalytic platforms,^72–76^ facilitating controlled C_α_-centered radical generation/stabilization, *in situ* ketonization, and subsequent reductive scission without intermediate isolation.

**Fig. 7:**
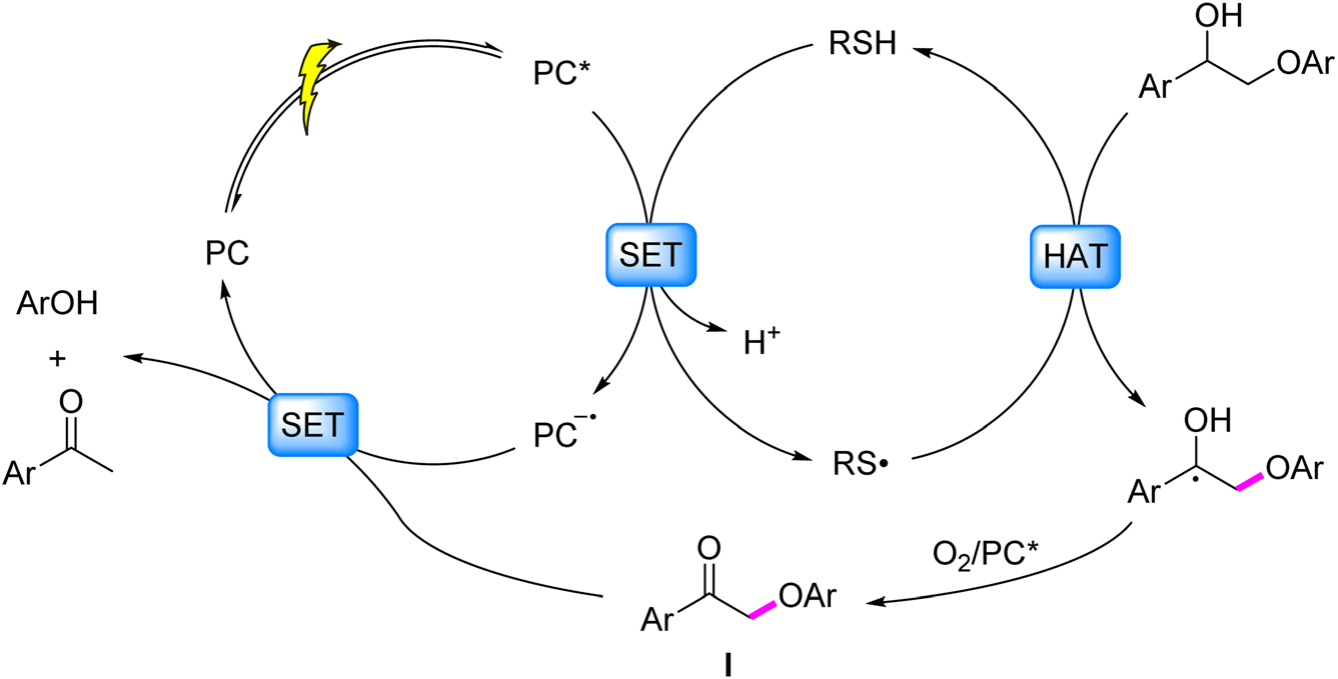
Mechanisms of C–O bond cleavage induced by C_α_-centered radicals generated *via* HAT.

Photocatalytic HAT-mediated C_β_–O bond cleavage in lignin proceeds through a radical cascade mechanism: under visible-light excitation, sulfur-based HAT reagents (*e.g.*, thiols/disulfides) undergo SET with the photocatalyst, followed by deprotonation to generate sulfur-centered radicals. These radicals selectively abstract hydrogen atoms from C_α_–H bonds to form C_α_-centered radicals, which are oxidized to ketonic intermediates with concomitant reduction of C_β_–O BDE. Subsequent SET between the ketonic intermediate and reduced photocatalyst drives C_β_–O bond scission (Fig. 7). A representative demonstration by Chen *et al.* (2019)^72^ employed a 4CzIPN/Ir-based catalytic system with NaOP(O)(OBu)_2_ as base and methyl thioglycolate as HAT reagent, achieving 91% acetophenone and 81% phenol yields from β-O-4 models.

**Fig. 8:**
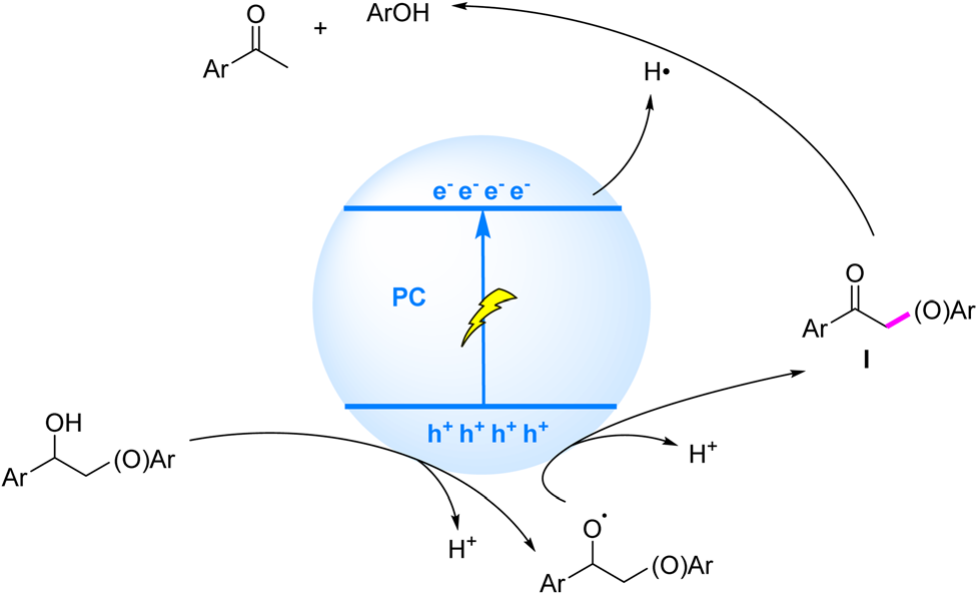
Mechanisms of C–O bond cleavage induced by ketonic intermediates generated *via* SET.

The photocatalytic mechanism for C_β_–O bond cleavage *via* ketonic intermediates, mediated by sequential SET processes, can be summarized as follows: under illumination, the substrate molecule undergoes oxidative electron transfer to the photocatalyst, followed by deprotonation to generate an oxygen-centered radical. This radical is further oxidized to form a ketonic intermediate, concomitant with weakening of the C_β_–O bond. Finally, the ketonic intermediate accepts an electron from the conduction band of the photocatalyst, triggering reductive cleavage of the C_β_–O bond (Fig. 8). Recent years have witnessed significant advancements in visible-light-driven heterogeneous photocatalytic systems for selective C_β_–O bond cleavage in lignin. Luo *et al.* (2017)^73^ pioneered a ZnIn_2_S_4_-based photocatalytic transfer hydrogenolysis system that ingeniously utilized the intrinsic C_α_H–OH moiety in β-O-4 structures as an endogenous hydrogen donor. Under optimized conditions, phenolic products from model compounds were obtained in 71–91% yields, while organosolv lignin conversion afforded *p*-hydroxyacetophenone derivatives in 10% yield. Mechanistic studies revealed that the process involved dehydrogenation of C_α_H–OH at the catalyst surface to form active hydrogen species, which subsequently facilitated efficient hydrogenolysis of adjacent C_β_–O bonds.

Progressively enhancing control, Han *et al.* (2019)^74^ developed Ni-modified ultrathin CdS nanosheets that enabled precise control over product selectivity through solvent modulation. In acetonitrile, β-O-4 model compounds were selectively oxidized to ketone intermediate I, which was further converted to benzoic acid and phenol. Remarkably, complete cleavage of β-O-4 models was achieved upon increasing the pH with 0.1 M KOH. Systematic investigations suggested that hydrogen species adsorbed on the catalyst surface played pivotal roles in bond cleavage. Further optimizing interfacial and electronic properties, Xu *et al.* (2023)^75^ designed a CdS-SH/TiO_2_ heterojunction system exhibiting exceptional performance. Surface thiol modification not only enhanced catalyst–substrate interaction but also optimized the band structure, with the constructed heterojunction significantly improving charge carrier separation efficiency. Under mild conditions, this system achieved 85% phenol yield and 87% acetophenone yield from model substrates. Most recently, Kumar *et al.* (2024)^76^ reported a CdS(3%)/TiO_2_ catalyst that pushed the reaction efficiency to new heights. The system achieved over 95% yield of monomeric products through an endogenous hydrogen transfer mechanism under blue light irradiation. Particularly noteworthy was its successful application in teak lignin conversion, delivering 24% yield of monomer derivatives.

### 3.2 HAT/SET-driven β-C–O cleavage *via* Cα radical intermediates

Photocatalytic generation of C_α_-centered radical intermediates can dramatically weaken the β-C–O bond dissociation energy from ∼55 kcal mol^−1^ to as low as ∼7.6 kcal mol^−1^, offering a promising strategy for C–O bond scission. However, the direct cleavage of the β-C–O bond in such radicals has long remained elusive due to challenges in stabilizing the radical species and controlling reaction selectivity. Recent breakthroughs have addressed this limitation: in homogeneous systems, HAT with spin center shift (SCS) mechanisms enable selective β-C–O bond activation,^77^ while heterogeneous systems achieve analogous transformations *via* electron–hole coupled (EHCO) mechanism.^78^

**Fig. 9:**
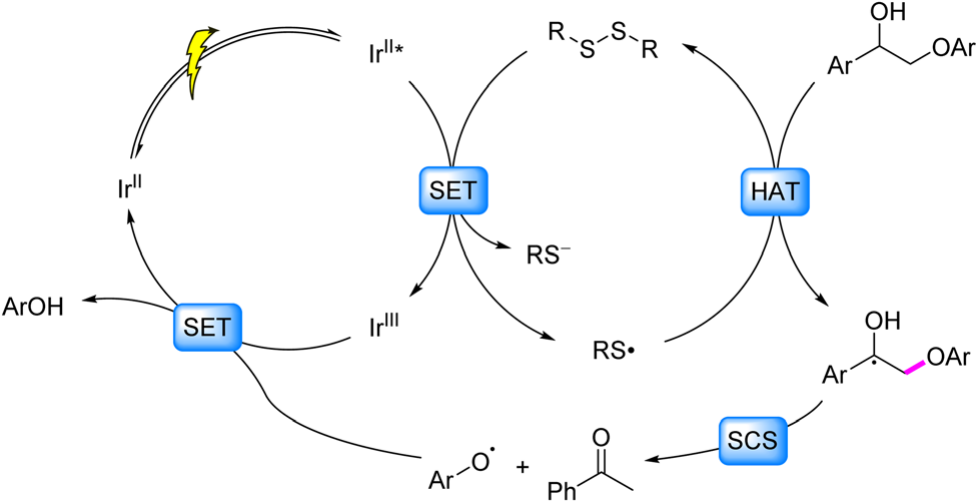
Mechanisms of C–O bond cleavage induced by C_α_ radicals generated *via* HAT.

Specifically illustrating the homogeneous strategy, Zhu *et al.* (2021)^77^ demonstrated a redox-neutral photocatalytic strategy employing disulfides as HAT reagents, achieving selective cleavage of β-O-4 model compounds with high efficiency (80% acetophenone and 73% phenol yield). Building upon this model system, the approach was further extended to solvolytic lignin from native pine, affording aromatic monomers in 2.26% yield (Fig. 9). Mechanistic studies revealed that the generated C_α_ radical intermediate undergoes SCS to trigger β-C–O bond cleavage, thereby selectively disrupting the most abundant β-O-4 linkage in lignin. Notably, the sulfur radical derived from cysteine-based HAT catalysts mediates the SCS process, resembling the bond-cleavage chemistry observed in ribonucleotide reductase active sites.

**Fig. 10:**
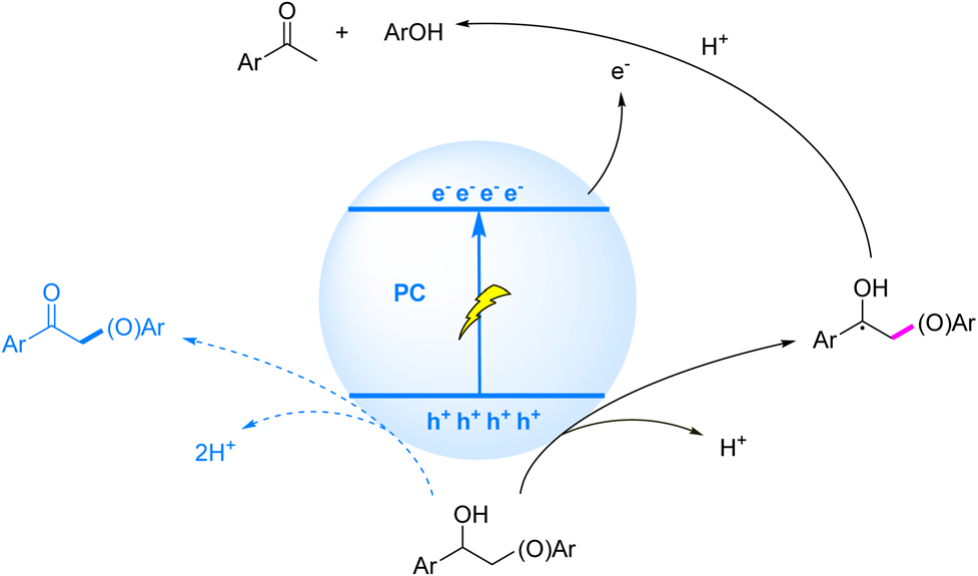
Mechanisms of C–O bond cleavage induced by C_α_ radicals generated *via* SET.

While HAT/SCS mechanisms have demonstrated remarkable efficacy in cleaving β-C–O bonds within homogeneous systems, heterogeneous photocatalysis *via* SET/EHCO processes has emerged as a compelling alternative strategy for lignin depolymerization. In the heterogeneous photocatalysis mechanism, photogenerated h^+^ selectively activate C_α_–H bonds to form crucial C_α_ radical intermediates, thereby weakening the adjacent β-C–O BDE, while conduction band electrons (e^−^) subsequently attack the activated β-C–O bond to achieve synergistic cleavage (Fig. 10).

This mechanistic framework was experimentally validated by Wu *et al.* in 2018,^78^ where CdS quantum dots (QDs) catalyzed visible-light-driven β-C–O bond cleavage in lignin β-O-4 model compounds *via* the EHCO pathway, achieving exceptional yields of acetophenone (∼91%) and phenol (∼93%). The success of this prototype system spurred subsequent interfacial engineering of CdS QDs to enhance catalytic performance. Crucially, the colloidal nature of QDs enables intimate contact with solid lignocellulose, selectively depolymerizing lignin while leaving cellulose/hemicellulose largely intact. When applied to birch lignin, the catalyst yielded 27 wt% aryl ketones *via* β-O-4 cleavage, establishing a robust strategy for selective bond scission under mild conditions that facilitates biomass valorization.

Building on this platform, Wu *et al.* (2019)^79^ demonstrated that the photocatalytic depolymerization efficiency of native lignin into functionalized aromatics directly correlates with ligand properties of CdS QDs. Using 3-mercaptopropionic acid-functionalized CdS–C3 QDs for technical lignin conversion, aromatic monomer yields showed positive correlation with substrate β-O-4 content, reaching 27 wt% under optimized conditions. Hydrophilic/hydrophobic balance modulation enabled stable colloidal dispersions, significantly enhancing QD-lignin interfacial contact and catalytic activity. Interfacial electron transfer was governed by ligand anchoring groups (–SH/–COOH) and alkyl chain lengths, with tunneling studies confirming exponential decay of charge transfer rates with increasing chain length.

Beyond ligand engineering, alternative catalyst design strategies have emerged. Notably, Yoo *et al.* (2020)^80^ demonstrated that employing Ag^+^-exchanged CdS as a photocatalyst significantly enhanced the efficiency and selectivity of C_β_–O bond cleavage in lignin β-O-4 model compounds by promoting electron transfer to the C_α_ radical intermediate, achieving near-quantitative conversion (>99%) under blue LED irradiation. The observed catalytic pathway aligns with the EHCO transfer mechanism. Although Ag^+^ exchange did not markedly alter the CdS band structure, it induced a Fermi level downshift, accelerating photogenerated charge carrier separation and enhancing proton affinity, thereby facilitating the EHCO transfer process.

Transcending the limitations of CdS-based photocatalysts, particularly their inherent toxicity and susceptibility to photo-corrosion, recent research has strategically expanded toward multinary sulfide architectures. These systems incorporate less toxic elements with tunable optoelectronic properties, enhancing the efficiency of the established EHCO mechanism for lignin valorization. This paradigm shift is exemplified by Lin *et al.*'s notable 2019 work,^81^ which demonstrated the photocatalytic activity of zinc indium sulfides (Zn_m_In_2_S_m+3_) for cleaving lignin β-O-4 linkages, achieving yields of acetophenone (86%) and phenol (82%). Precise tuning of Zn/In atomic ratios enabled strategic band structure engineering, with Zn_4_In_2_S_7_ exhibiting optimal β-O-4 scission performance under visible light. This superior performance originates from balanced visible-light harvesting and tailored redox potentials. The system afforded an 18.4 wt% yield of functionalized aromatic monomers from organosolv birch lignin. Mechanistic studies revealed that β-O-4 bond cleavage predominantly proceeds *via* a single-step redox-neutral pathway involving C_α_-centered radical intermediates, whereby C_β_–O bond scission is achieved through the EHCO mechanism.

Guided by this mechanistic understanding, subsequent catalyst engineering has markedly enhanced EHCO efficiency: Shao *et al.* (2022)^82^ reported Zn/S-rich ZnIn_2_S_4_ for visible-light-driven cleavage of C_β_–O bonds in lignin β-O-4 models. Using water as a green hydrogen source, near-quantitative conversion (>99%) and >90% total selectivity toward aromatic monomers (predominantly acetophenone and phenol) were achieved under mild conditions. Liu *et al.* (2023)^83^ constructed an S-scheme g-C_3_N_4_/Zn_4_In_2_S_7_ (CN/ZIS) heterojunction, achieving 99% selectivity for C_β_–O scission with near-complete substrate conversion in aqueous media. The stepwise charge-transfer channel enhanced carrier separation, yielding phenol (93.4%) and acetophenone (75.2%). The catalyst effectively depolymerized methoxy-modified β-O-4 analogs and native lignins (pine, wheat straw), significantly reducing molecular weights. Liu *et al.* (2024)^84^ developed a Z-scheme g-C_3_N_4_/CQD/CdZnS (CN/CQD/CZS) heterojunction *via* low-temperature hydrothermal synthesis. Carbon quantum dots (CQDs) acted as electron mediators, establishing an efficient Z-scheme pathway. Under optimized conditions, lignin depolymerization afforded vanillin and vanillic acid, with 84% model conversion and near-quantitative C_β_–O bond cleavage selectivity (>99%). Niu *et al.* (2025)^85^ engineered a hollow ZIF-8/CdS Z-scheme photocatalyst, achieving ultra-rapid C_β_–O bond cleavage with >99% chemoselectivity for phenol and acetophenone. Native lignins (alkali lignin, sodium lignosulfonate) were successfully depolymerized, with GC-MS confirming significant increases in aromatic monomers.

### 3.3 SET-driven cleavage of 4-O-5 and other aryl ether linkages

Addressing the challenge of high BDE in lignin-derived 4-O-5 C–O linkages, researchers have progressively overcome the bottleneck of mild cleavage through innovative photocatalytic systems and synergistic mechanisms. Current strategies focus on either generating highly reactive dearomatized carbon-centered radical intermediates *via* exogenous or *in situ* auxiliary agents to induce C–O bond scission, or achieving cleavage through cooperative SET and oxygen atom transfer (OAT) catalysis, with SET serving as the pivotal step.

**Fig. 11:**
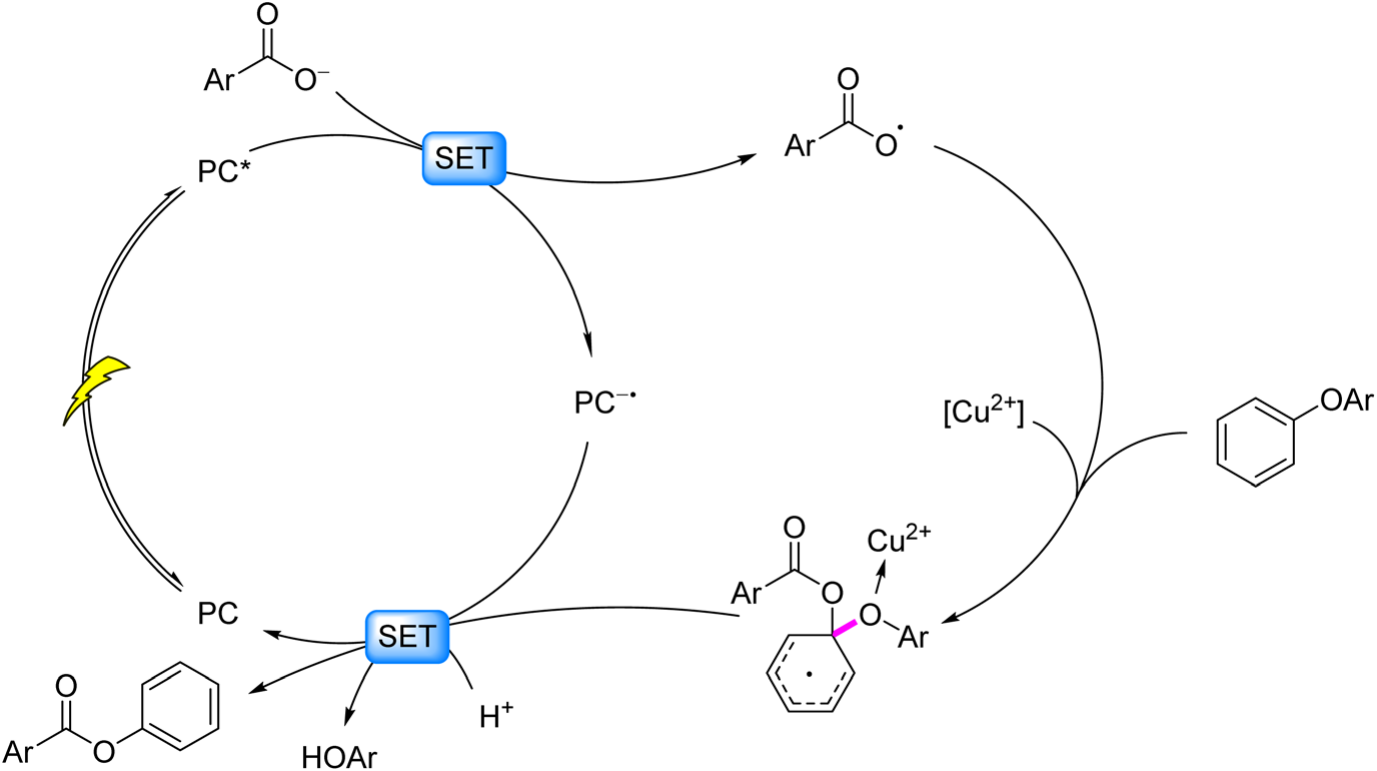
Photocatalytic acidolysis of C–O bonds in 4-O-5 models.

Wang *et al.* (2017)^86^ demonstrated visible-light-driven cleavage of 4-O-5 model compounds using acridinium salts or perylenediimide (PDI) photocatalysts at ambient temperature, wherein *ortho*-phenoxybenzoic acid substrates generated aryl carboxyl radicals for intramolecular electrophilic substitution. Catalytic base facilitated phenolate ester intermediate formation and subsequent hydrolysis to phenolic products, though this mechanism restricted applicability to carboxyl-functionalized 4-O-5 substrates. The team optimized the system in 2020 (ref. 87) by incorporating sterically hindered groups and electron-withdrawing aryl substituents at the acridinium 9-position. Synergized with copper(ii) salts, this modified photocatalyst enabled one-step 4-O-5 bond cleavage/hydrolysis *via* concerted intermolecular electrophilic substitution and SET at room temperature, achieving 80% phenol yield with 82% aryl carboxylic acid recovery. Mechanistically: visible light excites the photocatalyst (PC*) which undergoes reductive quenching by benzoate anions to produce PC^−^˙ and aryl carboxyl radicals; diaryl ether substrates trap these radicals forming reactive intermediates; copper(ii) mediates SET between intermediates and PC^−^˙ while acting as a Lewis acid to activate the C–O bond, ultimately cleaving the 4-O-5 linkage to yield phenyl benzoate and phenol (Fig. 11).

**Fig. 12:**
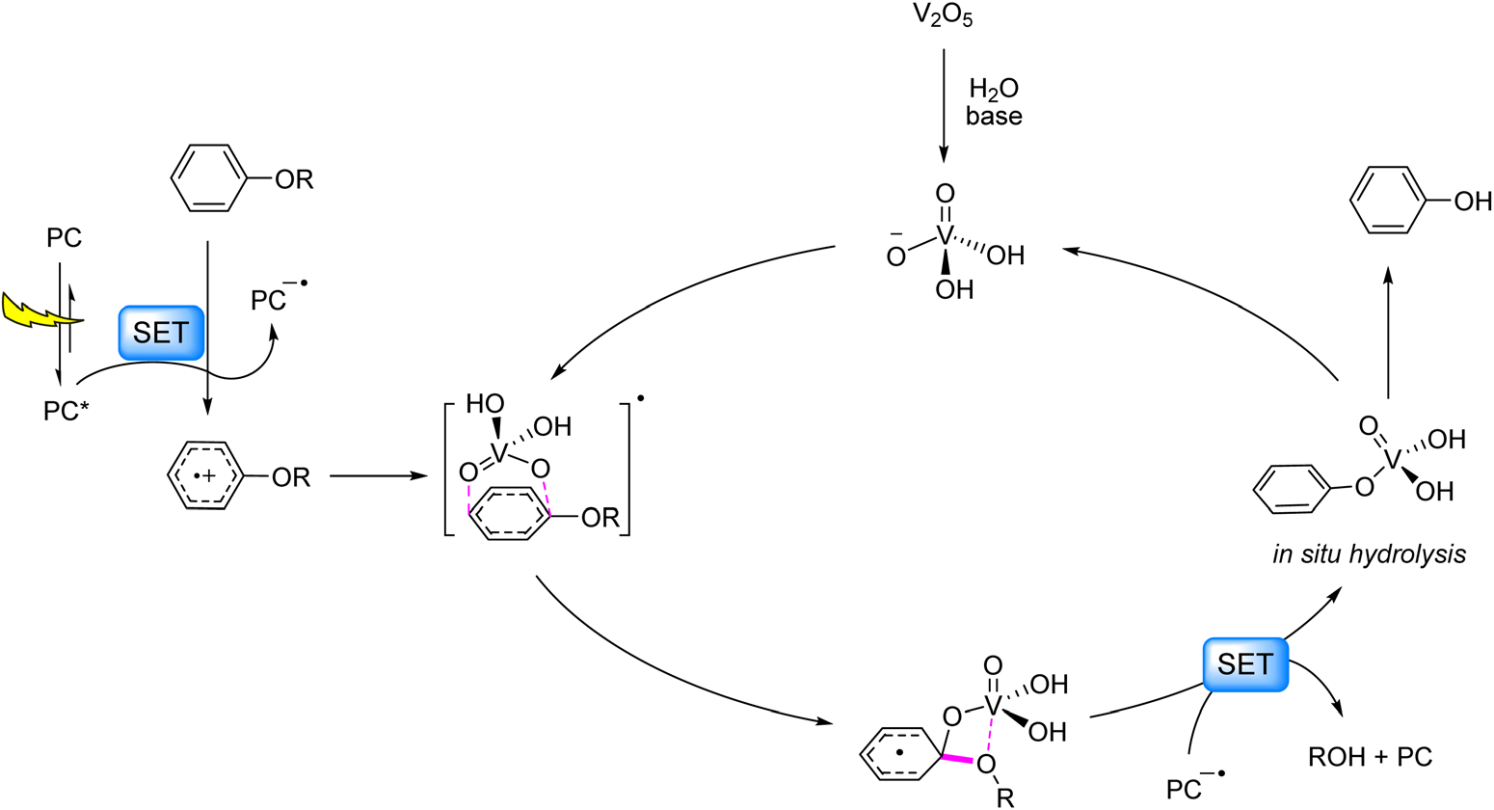
Photocatalytic hydrolysis of C–O bonds in 4-O-5 models.

The ideal strategy for 4-O-5 C–O bond cleavage lies in single-step hydrolysis at ambient temperature. In 2023, the same group realized this objective by employing their developed acridinium-based photocatalyst in synergy with V_2_O_5_, enabling light-driven hydrolysis of diaryl ethers to produce two phenolic molecules.^88^ Mechanistic studies revealed that the diaryl ether reductively quenches the PC*, generating an aryl cation radical and reduced PC^−^˙. Concurrently, V_2_O_5_ generates H_2_VO_4_^−^*in situ* under alkaline conditions, which nucleophilically attacks the diaryl ether cation radical to form a Meisenheimer-like intermediate. Spin density redistribution within this intermediate, coupled with vanadium–oxygen interactions, triggers C–O bond scission, yielding phenyl vanadate and an oxygen-centered radical. The radical is quenched to form phenol or alcohol, while phenyl vanadate undergoes hydrolysis to regenerate phenol and H_2_VO_4_^−^, thereby closing the catalytic cycle (Fig. 12). This vanadium-photocatalyst cooperative system successfully cleaved 4-O-5, α-O-4, and β-O-4 linkages, demonstrating broad applicability in lignin valorization.

**Fig. 13:**
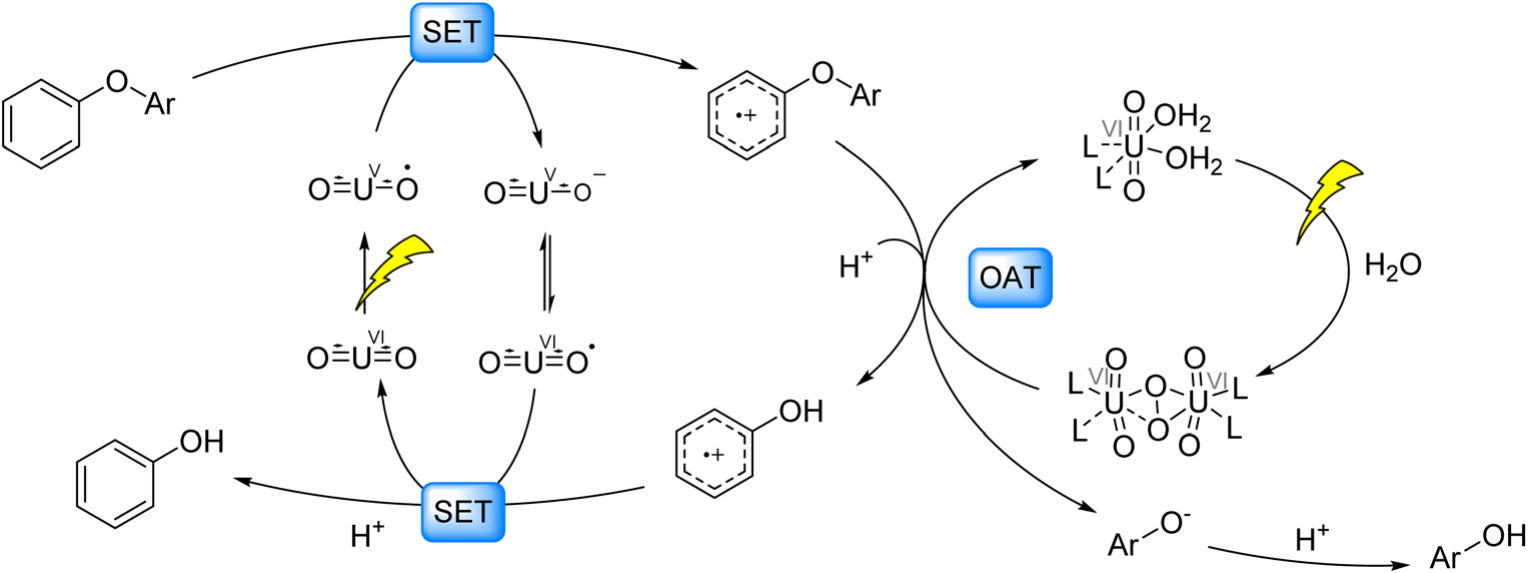
Photocatalytic cleavage of C–O bonds in 4-O-5 models.

Parallel Developments: Zhou *et al.* (2022)^89^ reported UO_2_(NO_3_)_2_·6H_2_O as a photocatalyst for acidic hydrolysis of diaryl ethers to phenolic products. Key mechanistic steps involve reductive quenching of the uranyl cation (UO_2_˙) by the diaryl ether, generating an aryl cation radical. Subsequent OAT between the diaryl ether cation radical and uranyl peroxide species incorporates water-derived oxygen atoms, forming an intermediate aryl cation radical and phenoxide anion. The aryl cation radical undergoes single-electron oxidation by UO_2_, yielding phenol, while the phenoxide anion combines with protons to produce additional phenol (Fig. 13).

Photocatalytic cleavage of C–O bonds in α-O-4 model compounds primarily relies on external hydrogen donors (*e.g.*, ethanol, isopropanol) for hydrogenolysis. Key advances encompass: Han *et al.* (2019)^90^ constructed a composite catalyst by co-immobilizing Ni^2+^ complexes with plasmonic nanoparticles (Au or Ag NPs) on γ-Al_2_O_3_. Systems containing 2.5 wt% Au NPs or 4.3 wt% Ag NPs achieved efficient C–O bond cleavage under visible light, affording 98% conversion (2.5Au-ASN-Ni^2+^) and 96% conversion (4.3Ag-ASN-Ni^2+^) with ∼50% phenol selectivity while suppressing aromatic ring over-hydrogenation. Mechanistic studies confirmed that Ni^0^ catalyzes C–O bond hydrogenolysis, with isopropanol (IPA) oxidation on plasmonic NPs providing hydrogen sources to yield phenol and toluene. Han *et al.* (2025)^91^ engineered a 2D heterojunction by integrating dipyrenylthio-perylenediimide (DPPDI) with TiO_2_. The Pd-loaded Pd@4%DPPDI/TiO_2_ achieved near-quantitative C–O bond cleavage (selectivity ≈100%) within 30 min, yielding toluene and phenol. The mechanism involves: enhanced charge separation at the heterojunction upon photoexcitation; ethanol oxidation by h^+^ releasing protons; Pd-captured e^−^ generating reactive H˙ species for selective hydrogenolysis of C–O bonds in α-O-4 model. This heterostructure broadens light absorption while suppressing charge recombination, significantly boosting photocatalytic efficiency.

## 4. Challenges and future perspectives

Despite the alluring prospects and encouraging progress, particularly with model compounds, in the photocatalytic valorization of lignin, its path towards practical implementation in biorefineries faces multiple critical challenges. Systematic identification and resolution of these bottlenecks are essential for achieving the efficient, high-value, and scalable utilization of lignin resources. This section will dissect the core challenges currently encountered and propose key future research priorities with reasoned expectations. The inherent complexity and heterogeneity of native lignin, comprising a network of diverse aromatic monomers linked by various bonds (*e.g.*, β-O-4, α-O-4, 4-O-5, 5–5, β-β, β-5), present a major obstacle to selective bond cleavage. Its branched structure, diverse functional groups (methoxy, hydroxyl, *etc.*), and uneven molecular weight distribution further complicate selective depolymerization. While extensive research on simplified model compounds, especially β-O-4 dimers, has advanced mechanistic understanding, these models fail to fully replicate the recalcitrance of native lignin polymers arising from steric hindrance, bond energy distributions, and complex intermolecular/intramolecular interactions. Consequently, catalysts optimized for model substrates often suffer from incomplete degradation, low target monomer yields, and significant side reactions (*e.g.*, over-oxidation, C–C bond cleavage, repolymerization) when applied to real lignin. Therefore, there is an urgent need to develop robust and adaptive catalytic systems capable of effectively recognizing and preferentially cleaving specific bond types (notably the abundant yet relatively weak β-O-4 linkage) while tolerating lignin's intrinsic variability.

Furthermore, compatibility issues between photocatalytic systems and raw biomass feedstocks pose significant hurdles. The opaque or semi-opaque nature of native lignin feedstocks and their biomass matrices (*e.g.*, untreated lignocellulose) severely impedes effective light penetration and uniform distribution, leading to large light intensity gradients within reactors and low photon utilization efficiency. Compounding this issue, lignin itself contains abundant chromophores (*e.g.*, phenolic hydroxyls, conjugated structures) with strong absorption in the UV-visible region. These chromophores compete with the photocatalyst for excitation light, significantly reducing catalytic excitation efficiency and potentially triggering non-catalytic photodegradation side reactions. Thus, enhancing the applicability of photocatalytic systems to raw/untreated lignin feedstocks by overcoming light transmission and competitive absorption challenges is a prerequisite for practical deployment. Additionally, scalability and process economics are constrained by several bottlenecks: reliance on energy-intensive, high-power UV or specific-wavelength visible light sources increases costs and hinders large-scale feasibility; insufficient catalyst stability under prolonged, potentially harsh reaction conditions (*e.g.*, strong oxidizing environments, inhibitors) leads to deactivation *via* photocorrosion, leaching, or structural degradation, necessitating frequent replacement/regeneration; and the inherent difficulty in efficiently and cost-effectively separating and purifying target high-value chemicals (*e.g.*, specific phenolic monomers) from the complex mixture of aromatic monomers, oligomers, and oxygenated small molecules produced during photocatalytic lignin degradation represents a major engineering challenge. Addressing these requires the development of low-energy lighting solutions, highly stable/long-lived catalysts, and efficient, economical product separation strategies to assess techno-economic feasibility and enable scale-up.

To effectively overcome these challenges and propel photocatalytic lignin conversion from the laboratory towards industrialization, future research should concentrate on several interconnected key directions. Crucially, the development of intelligent catalytic systems for complex substrates is paramount. This includes designing multifunctional photocatalysts or cascade catalytic systems that integrate efficient, selective scission of specific bonds (*e.g.*, β-O-4) with the *in situ* stabilization/protection of key intermediates (*e.g.*, radicals). For instance, introducing cocatalysts or functional groups that rapidly capture active radicals and direct them towards target products could effectively suppress detrimental repolymerization (condensation) and over-degradation side reactions, significantly improving the efficiency of obtaining selective monomers from native lignin. Complementing this, “biomimetic” and computation-aided design, leveraging computational chemistry (DFT, MD, *etc.*) and machine learning to simulate lignin fragment interactions with catalyst active sites, will be vital. This approach enables precise tuning of catalyst redox potentials, band structures, surface properties, and substrate binding pockets to better accommodate lignin's structural irregularity and heterogeneity, accelerating the discovery of efficient, broad-spectrum catalysts. Meanwhile, constructing photonic hybrid catalytic systems offers significant potential. Actively exploring synergistic coupling of photocatalysis with other processes (*e.g.*, enzymatic, electrochemical, thermochemical, sonochemical) – such as photobiocatalytic cascades using photocatalytic pretreatment to open the lignin macromolecular structure or activate recalcitrant bonds (*e.g.*, C–C) followed by highly selective lignin-degrading enzymes (*e.g.*, laccases, peroxidases) for fine cleavage and product control, or photoelectrocatalysis utilizing electric fields to modulate charge carrier separation efficiency and reaction pathways for potential potential-dependent selective cleavage – can overcome limitations of single-mode photocatalysis, enhance the ability to handle recalcitrant linkages, improve overall selectivity, and potentially reduce energy consumption.

Concurrently, optimizing photon delivery and reactor engineering is essential for scalability. Prioritizing the development of efficient photoreactors suitable for lignin suspensions or solid–liquid mixtures is necessary. Strategies include exploring continuous flow systems (enhancing throughput and mass/light transfer), employing light-scattering/guiding materials (*e.g.*, optical fiber reactors, scattering particle beds), and designing specialized light source configurations (*e.g.*, internal illumination, multi-source arrays) to maximize photon penetration depth and utilization efficiency in turbid media. Furthermore, enhancing direct solar light utilization by developing narrow-bandgap photocatalysts responsive to visible or even near-infrared light (*e.g.*, engineered polymeric carbon nitride, dye-sensitized materials, plasmonic metal/semiconductors) and optimizing reactor design for effective solar energy harvesting is fundamental for reducing process energy demands. Finally, enhancing catalyst stability and process sustainability is critical. Advanced catalyst engineering should focus on materials with intrinsic high stability or self-healing capabilities, such as covalent organic frameworks (COFs), metal–organic frameworks (MOFs), or catalysts covalently immobilized on stable supports (*e.g.*, carbon materials, ceramics, polymers), to improve mechanical robustness and resistance to photocorrosion and chemical attack, alongside exploring smart nanomaterials with self-healing properties. Moreover, realizing efficient catalyst recovery and reuse (*e.g.*, *via* magnetic separation, membrane separation, fixed-bed designs) is key to lowering costs and environmental impact. Research into recovering precious metals from spent catalysts and implementing green alternatives and recycling loops for solvents/additives will further enhance the overall process sustainability, aligning with green chemistry and circular bioeconomy principles.

## 5. Conclusions

Photocatalysis has emerged as a compelling strategy for unlocking the intrinsic value of lignin, offering a sustainable pathway to valorize this abundant yet underutilized biopolymer into high-value aromatic chemicals under mild conditions. Significant progress has been achieved, particularly in elucidating reaction mechanisms using model compounds and developing novel photocatalysts capable of cleaving dominant linkages like β-O-4. However, this review underscores that bridging the gap between promising laboratory results and industrial implementation requires overcoming persistent, interconnected challenges. The inherent structural complexity and heterogeneity of native lignin—encompassing diverse bond types, branching patterns, and functional groups—remain a fundamental barrier to achieving high selectivity and yield in real biomass feedstocks. This complexity, coupled with limitations in light penetration through opaque matrices, competitive photon absorption by lignin chromophores, catalyst instability under prolonged operation, and the energy-intensive nature of current processes, constrains practical scalability and economic viability.

To realize the full potential of photocatalytic lignin valorization within integrated biorefineries, future research must adopt a holistic and adaptive approach. First, catalyst design must evolve beyond model systems. Leveraging computational modeling and machine learning to engineer robust, multifunctional photocatalysts—tailored to recognize specific linkages within lignin's irregular architecture while suppressing undesirable side reactions—is paramount. Second, synergistic hybrid processes integrating photocatalysis with enzymatic, electrochemical, or mild thermochemical steps offer a powerful strategy to enhance selectivity, address recalcitrant bonds, and improve overall energy efficiency. Third, innovations in photoreactor engineering (*e.g.*, advanced flow systems, optimized light management for turbid media) and the development of visible/near-infrared-active, solar-responsive photocatalysts are critical to maximize photon utilization efficiency and enable scalability. Finally, ensuring long-term sustainability necessitates a strong focus on catalyst stability through novel material designs (*e.g.*, MOFs, COFs, immobilized systems), efficient recycling protocols, and the adoption of green solvents aligned with circular economy principles. By systematically addressing these priorities, photocatalytic technology can transform lignin from a waste stream into a cornerstone renewable resource for sustainable chemical production, contributing significantly to the transition towards a circular bioeconomy.

## Author contributions

F.-F. Tan conceived the study, designed and performed the research, wrote the original draft, and reviewed and edited the manuscript. The author also supervised the project, administered it, and acquired funding. F.-F. Tan has read and agreed to the published version of the manuscript.

## Conflicts of interest

There are no conflicts to declare.

## Data Availability

No primary research results, software or code have been included and no new data were generated or analysed as part of this review.
